# Machine Learning Methods Using Artificial Intelligence Deployed on Electronic Health Record Data for Identification and Referral of At-Risk Patients From Primary Care Physicians to Eye Care Specialists: Retrospective, Case-Controlled Study

**DOI:** 10.2196/48295

**Published:** 2024-03-12

**Authors:** Joshua A Young, Chin-Wen Chang, Charles W Scales, Saurabh V Menon, Chantal E Holy, Caroline Adrienne Blackie

**Affiliations:** 1 Department of Ophthalmology New York University School of Medicine New York, NY United States; 2 Data Science Johnson & Johnson MedTech Raritan, NJ United States; 3 Medical and Scientific Operations Johnson & Johnson Medtech, Vision Jacksonville, FL United States; 4 Mu Sigma Business Solutions Private Limited Bangalore India; 5 Epidemiology and Real-World Data Sciences, Johnson & Johnson MedTech New Brunswick, NJ United States; 6 Medical and Scientific Operations Johnson & Johnson MedTech, Vision Jacksonville, FL United States

**Keywords:** decision support for health professionals, tools, programs and algorithms, electronic health record, primary care, artificial intelligence, AI, prediction accuracy, triaging, AI model, eye care, ophthalmic

## Abstract

**Background:**

Identification and referral of at-risk patients from primary care practitioners (PCPs) to eye care professionals remain a challenge. Approximately 1.9 million Americans suffer from vision loss as a result of undiagnosed or untreated ophthalmic conditions. In ophthalmology, artificial intelligence (AI) is used to predict glaucoma progression, recognize diabetic retinopathy (DR), and classify ocular tumors; however, AI has not yet been used to triage primary care patients for ophthalmology referral.

**Objective:**

This study aimed to build and compare machine learning (ML) methods, applicable to electronic health records (EHRs) of PCPs, capable of triaging patients for referral to eye care specialists.

**Methods:**

Accessing the Optum deidentified EHR data set, 743,039 patients with 5 leading vision conditions (age-related macular degeneration [AMD], visually significant cataract, DR, glaucoma, or ocular surface disease [OSD]) were exact-matched on age and gender to 743,039 controls without eye conditions. Between 142 and 182 non-ophthalmic parameters per patient were input into 5 ML methods: generalized linear model, L1-regularized logistic regression, random forest, Extreme Gradient Boosting (XGBoost), and J48 decision tree. Model performance was compared for each pathology to select the most predictive algorithm. The area under the curve (AUC) was assessed for all algorithms for each outcome.

**Results:**

XGBoost demonstrated the best performance, showing, respectively, a prediction accuracy and an AUC of 78.6% (95% CI 78.3%-78.9%) and 0.878 for visually significant cataract, 77.4% (95% CI 76.7%-78.1%) and 0.858 for exudative AMD, 79.2% (95% CI 78.8%-79.6%) and 0.879 for nonexudative AMD, 72.2% (95% CI 69.9%-74.5%) and 0.803 for OSD requiring medication, 70.8% (95% CI 70.5%-71.1%) and 0.785 for glaucoma, 85.0% (95% CI 84.2%-85.8%) and 0.924 for type 1 nonproliferative diabetic retinopathy (NPDR), 82.2% (95% CI 80.4%-84.0%) and 0.911 for type 1 proliferative diabetic retinopathy (PDR), 81.3% (95% CI 81.0%-81.6%) and 0.891 for type 2 NPDR, and 82.1% (95% CI 81.3%-82.9%) and 0.900 for type 2 PDR.

**Conclusions:**

The 5 ML methods deployed were able to successfully identify patients with elevated odds ratios (ORs), thus capable of patient triage, for ocular pathology ranging from 2.4 (95% CI 2.4-2.5) for glaucoma to 5.7 (95% CI 5.0-6.4) for type 1 NPDR, with an average OR of 3.9. The application of these models could enable PCPs to better identify and triage patients at risk for treatable ophthalmic pathology. Early identification of patients with unrecognized sight-threatening conditions may lead to earlier treatment and a reduced economic burden. More importantly, such triage may improve patients’ lives.

## Introduction

In the United States alone, more than 93 million adults were at high risk for vision loss in 2017; however, only 56.9% visited an eye care professional annually, and only 59.8% received a dilated eye examination [1]. More than 4 million Americans suffer from uncorrectable vision impairment, and more than 1 million are blind; this number is predicted to more than double by 2050 to 9 million due to the increasing epidemics of diabetes and other chronic diseases and our rapidly aging US population [2]. The impact of poor eyesight is manifest in its potentiation of comorbidities, particularly in increasing the risk of disability in patients with cognitive impairment [3]. Early identification of patients with unrecognized sight-threatening conditions may lead to earlier treatment and a reduced economic burden. More importantly, such triage may improve patients’ lives.

The identification and referral of patients at risk of vision loss from primary care practitioners (PCPs) to eye care professionals remains a challenge [4]. A 2010 study identified a number of barriers, including a lack of access to ophthalmic screening within the setting of the PCP’s office [4]. Some regional efforts have been made to improve the efficiency of triage of patients at risk for glaucoma [5] and diabetic retinopathy (DR) [6]; however, existing initiatives triage patients on only a few demographic and comorbidity parameters, whereas many systemic associations have been identified for age-related macular degeneration (AMD), cataract, DR, glaucoma, and ocular surface disease (OSD) [7-16].

Artificial intelligence (AI) modeling techniques are becoming increasingly important in ophthalmology in particular and medicine in general [17-20]. In ophthalmology, AI is used to calculate intraocular lens (IOL) powers [21-23], predict glaucoma progression [24,25], recognize DR [26], and classify ocular tumors [27]. To the best of our knowledge, AI has not yet been used to triage primary care patients for ophthalmology referral. In this study, the development, validation, and testing of multiple predictive machine learning (ML) methods for 5 leading sight-threatening and treatable ocular pathologies (ie, AMD, visually significant cataract, DR, glaucoma, and OSD) that have the potential to be used by PCPs to triage patients, based on existing data in their electronic health records (EHRs), for referral to eye care specialists were reported.

## Methods

### AI Modeling

All AI techniques have in common the process of “training,” the adjustment of importance (ie, weights) of attributes or intermediate values, based on a set of data referred to as a training set. The model performance is then assessed against another set of data called the test set. Similar model performance on training and test sets demonstrates model generalizability. The advent of large clinical databases has made possible the construction and training of both ML and neural network AI models. To this end, a large commercial EHR database that includes demographic, diagnostic, and therapeutic data to create and curate an ophthalmologically focused data set from which predictive models of multiple eye diseases can be built was used. We chose to compare several different ML methods to create models that might be used by PCPs to triage patients for referral to an eye care specialist. The models thus created used non-ophthalmic clinical and demographic data to assess relative risk scores for AMD, cataract, DR, glaucoma, and OSD.

### Data Source

This retrospective, case-controlled study used data from the Optum deidentified EHR data set. EHRs provide efficient access to detailed patient-level longitudinal data that represent integral components of clinical care that may not necessarily be available through other retrospective database sources, such as administrative claims databases or patient registries [28,29]. The Optum EHR data set consists of data primarily from the United States and represents the clinical information of more than 80 million patients, including at least 7 million patients in each US census region from May 2000 to December 2019. Data from multiple EHR platforms, including Cerner, Epic, GE, and McKesson, are analyzed by Optum by means of natural language processing (NLP) to extract information about patient demographics, enrollment, diagnoses, biometrics, laboratory results, procedures, and medications [30]. The data set draws upon a network of more than 140,000 providers at more than 700 hospitals and 7000 clinics.

### Ethical Considerations

The use of the Optum EHR data set was reviewed by the New England Institutional Review Board (IRB) and was determined to be exempt from broad IRB approval as this research project did not involve human subject research.

### Outcome Measures

This study sought to predict the diagnosis of 5 major eye pathologies: AMD, cataract, DR, glaucoma, and OSD. The classification of AMD was based on the *International Classification of Diseases, 10th Revision* (ICD-10) codes and subdivided into nonexudative (H35.31%) and exudative (H35.32%) groups, in which “%” represents a wildcard. The classification of cataract required a more restrictive definition than simply H25%. Since no ICD-10 code distinguishes visually significant cataracts from those of lesser impact, we chose to use cataract surgery as a surrogate for visually significant cataract. For this study, cataract was defined by the cataract surgery Current Procedural Terminology (CPT) codes of 66982 and 66984 rather than by ICD-10. The classification of DR was based on the data set ICD-10 codes and subdivided into type 1 nonproliferative diabetic retinopathy (NPDR; H10.31%-H10.34%), type 1 proliferative diabetic retinopathy (PDR; H10.35%), type 2 NPDR (H11.31%-H11.34%), and type 2 PDR (H11.35%). Glaucoma was defined by the presence of 1 or more of 3 criteria: an ICD-10 code of H40.1% (open-angle glaucoma), the prescription of glaucoma medication, or the presence of a CPT code indicating glaucoma surgery. This definition was developed to capture not only patients with a recorded diagnosis of glaucoma but also those patients being treated for glaucoma or high-risk ocular hypertension for whom the diagnosis of glaucoma was not recorded in the data set. Similar to cataract, OSD required narrower criteria than simply H04.1% and H02.88% since these codes do not distinguish OSD requiring treatment from more mild presentations. For this study, OSD was defined rather restrictively as patients receiving cyclosporine ophthalmic emulsion 0.05%, cyclosporine ophthalmic solution 0.09%, or lifitegrast ophthalmic solution 5% (see Tables 1 and 2).

**Table 1:** Listed medications for glaucoma.

Type of medication	Examples
Beta blockers	Levobunolol (Betagan, Akbeta), timolol (Timoptic, Betimal, Istalol), carteolol (Ocupress), metipranolol (Optipranolol), timolol gel (Timoptic Xe), betaxolol (Betoptic, Betoptic S)
Alpha agonists	Apraclonidine (Iopidine), brimonidine (Alphagan, Alphagan P), dipivefrin (Propine)
Carbonic anhydrase inhibitors	Dorzolamide (Trusopt), brinzolamide (Azopt)
Prostaglandin analogs	Latanoprost (Xalatan), bimatoprost 0.01% (Lumigan), travoprost (Travatan Z), tafluprost (Zioptan), latanoprostene bunod (Vyzulta)
Prostaglandin analogs (combined medications)	Dorzolamide/timolol (Cosopt and Cospot Pf), brimonidine/timolol (Combigan), brinzolamide/brimonidine (Simbrinza), netarsudil/latanoprost (Rocklatan)
Rho kinase inhibitors	Netarsudil (Rhopressa)

**Table 2:** Listed procedures for glaucoma.

ICD-10^a^ code	Description
0191T	Insertion of anterior segment aqueous drainage device, without extraocular reservoir, internal approach, into the trabecular meshwork; initial insertion
0253T	Insertion of anterior segment aqueous drainage device, without extraocular reservoir, internal approach, into the suprachoroidal space
0376T	Insertion of anterior segment aqueous drainage device, without extraocular reservoir, internal approach, into the trabecular meshwork; each additional device insertion (list separately in addition to code for primary procedure)
0449T	Insertion of aqueous drainage device, without extraocular reservoir, internal approach, into the subconjunctival space; initial device
0450T	Insertion of aqueous drainage device, without extraocular reservoir, internal approach, into the subconjunctival space; each additional device (list separately in addition to code for primary procedure)
0474T	Insertion of anterior segment aqueous drainage device, with creation of intraocular reservoir, internal approach, into the supraciliary space
65820	Goniotomy
65855	Trabeculoplasty laser
66174	Transluminal dilation of aqueous outflow canal; without retention of device or stent
66175	Transluminal dilation of aqueous outflow canal; with retention of device or stent
66179	Aqueous shunt to extraocular equatorial plate reservoir, external approach; without graft
66180	Aqueous shunt to extraocular equatorial plate reservoir, external approach; with graft
66183	Insertion of anterior segment aqueous drainage device, without extraocular reservoir, external approach
66184	Revision of aqueous shunt to extraocular equatorial plate reservoir; without graft
66185	Revision of aqueous shunt to extraocular equatorial plate reservoir; with graft
66710	ciliary body destruction by cyclophotocoagulation, trans-scleral approach
66711	ciliary body destruction by cyclophotocoagulation, endoscopic approach (endoscopic cyclophotocoagulation)

^a^ICD-10: International Classification of Diseases, 10th Revision.

### Creation of Patient Cohorts

Five distinct cohorts (ocular cohorts) of patients (AMD n=294,739, cataract n=1,191,492, DR n=348,056, glaucoma n=843,560, and OSD n=660,218) were selected from the Optum EHR data set based on the aforementioned code definitions from October 2015 onward (to limit the analysis to the start of the ICD-10 coding system in the United States). The inclusion criteria were as follows: patients with diagnosis codes such as H3530%/H3531%/H3532%, H25%, E083%/E093%/E103%/E113%/E133%, H40%, or H041%/H0288% and EHRs with an ICD-10 diagnosis code type. Patients were excluded if they had an unknown birth year, were younger than 15 years, had less than 60 days of continuous enrollment in the database prior to their diagnosis, had a gender labeled as unknown, or had undergone a cataract-related procedure or diagnosis at baseline or not undergone a cataract-related procedure and diagnosis in the follow-up. Patients with multiple conditions (eg, glaucoma and OSD) were identified in both the glaucoma and OSD cohorts. For each patient, demographic information, complete clinical and drug use information, and comorbidities were identified. Multimedia Appendix 1 presents the patient inclusion and exclusion criteria and attrition data. All patients with the diagnoses present in the database during the specified inclusion period were considered for inclusion. Finally, the patients were segregated into subsets based on the AMD subtype or the DR subtype. In addition, only those patients who had open-angle glaucoma, had consumed a glaucoma-related medication, had undergone a glaucoma-related procedure in the follow-up, or had consumed dry eye and meibomian gland dysfunction (DEMGD)–related medications in the follow-up were retained. The final cohorts were as follows: exudative AMD n=32,072 (10.9%), nonexudative AMD n=114,839 (39%), cataract n=197,570 (16.6%), type I NPDR n=20,654 (5.9%), type I PDR n=4465 (1.3%), type II NPDR n=155,927 (44.8%), type II PDR n=21,032 (6%), glaucoma n=192,727 (22.8%), and OSD n=3720 (0.6%).

For each of the 5 cohorts, a control population was created from the pool of patients without ocular conditions. The control populations were matched 1:1 to each ocular cohort using exact matching on age and gender. A total of 743,039 patients with AMD, visually significant cataract, DR, glaucoma, or OSD were available in the Optum deidentified EHR data set, so these were exact-matched on age and gender to 743,039 controls without eye conditions.

### Machine Learning

Several distinct ML approaches were followed to model the outcomes described earlier. These included the generalized linear model (GLM) [31], L1-regularized logistic regression (L1-LR) [32], random forest (RF) [33], Extreme Gradient Boosting (XGBoost) [34], and J48 decision tree (DT) [35].

### Data Preprocessing

The data set consisted of 380 attributes, including demographic information, diagnoses, biometrics, laboratory results, procedures, and medications. Since some of these attributes, particularly some of the laboratory tests, were only sparsely represented, the data were pruned to remove attributes (ie, “features” in ML) with more than 20% missing values. Missing values were imputed with medians for continuous variables (eg, BMI), with a “Missing” group for categorical variables (eg, smoke or alcohol usage), and with the most frequent value for binary variables (eg, levels of lab test results). Winsorization of the data was performed to remove outliers and replace these with 0.1 and 99.9 percentile values. Further feature engineering was performed to remove or combine highly correlated features, such as “rheumatoid arthritis/collagen vascular disease” and its highly correlated cognate “connective tissue disease.” These feature engineering steps were performed individually for each case-controlled data set of each subpathology. The resultant data sets exhibited between 142 and 182 features after the above-described culling. The feature exclusion data sets for each of the 9 subpathologies were modeled using each of 5 distinct modeling strategies to produce a total of 45 individual ML models. These 45 models were produced and compared in a competitive fashion to identify the single-best model for each pathology.

### Model Strategies

Logistic regression without regularization (LR), L1-LR, RF, and XGBoost models were performed in Python (3.8.5) using the Scikit-learn (0.23.2) and XGBoost (1.2.0) libraries. Next, 80% of the data were used for training, and 20% of the data were used for testing with 5-fold cross-validation. A grid search was used to optimize hyperparameters. For L1-LR, the regularization strength C was tuned. In the RF algorithm, the space of the number of trees and the maximum depth of each tree combination were searched. The hyperparameter tuning for XGBoost included the learning rate and the maximum depth of each tree. The ML modeling pipeline was established, and information of missing values fit and learned from the training data was applied to the test data set to avoid information leakage. J48 DT modeling, a Java-based implementation of the C4 tree, was performed in the WEKA ML workbench (University of Waikato). Finally, 10-fold cross-validation was used with an initial leaf size of 2% of the data set. The area under the curve (AUC) was assessed for all algorithms for each outcome to measure the overall performance of the binary classification models.

## Results

### Cohort Details

The demographic information of each cohort is shown in Table 3. Briefly, the total populations for modeling, for each cohort, varied in size from 7440 to 395,140. Populations were mostly female for AMD, cataract, glaucoma, and OSD requiring medications, and the average age ranged from 51 to 80 years.

**Figure 1:**
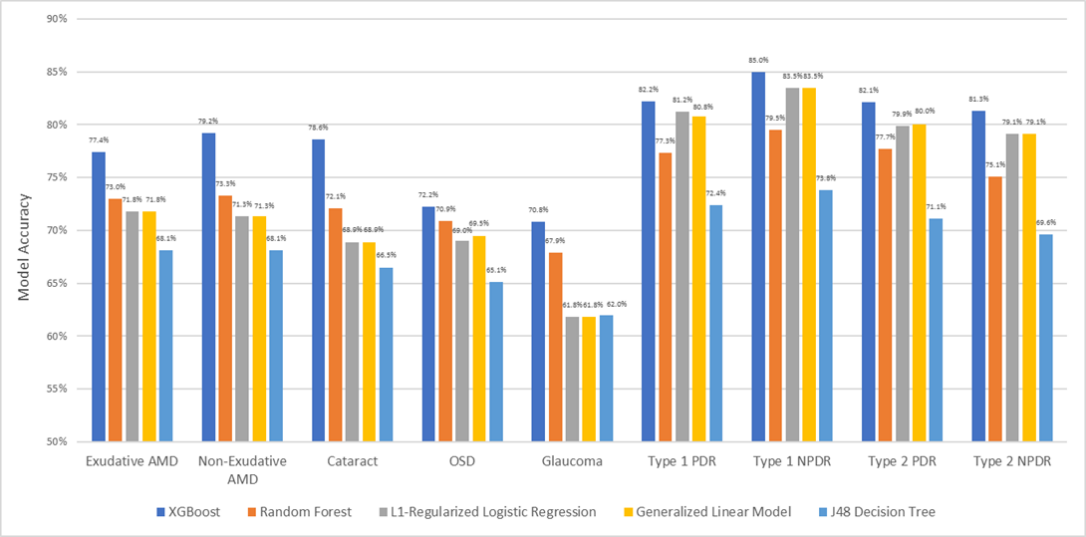
Model accuracy by pathology degeneration; AUC = area under the curve; CI = confidence interval; J48 = Decision tree; LR = Logistic Regression without regularization; LR-L1 = L1-regularized logistic regression; NPDR = non-proliferative diabetic retinopathy; OSD = ocular surface disease; PDR = proliferative diabetic retinopathy; XGB = XGBoost.

**Figure 2:**
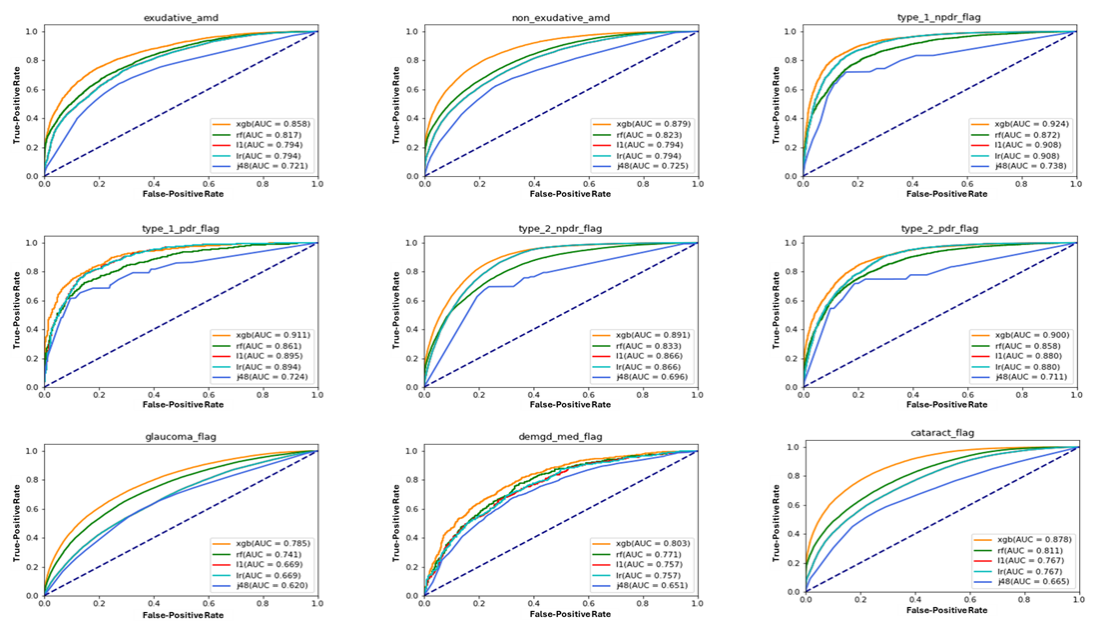
Receiver operating characteristic (ROC) curves illustrating the diagnostic ability of the models for the 9 pathologies. amd: age-related macular degeneration; auc: area under the curve; demgd: dry eye and meibomian gland dysfunction; j48: decision tree; l1: L1-regularized logistic regression; lr: logistic regression without regularization; npdr: nonproliferative diabetic retinopathy; pdr: proliferative diabetic retinopathy; rf: random forest; xgb: Extreme Gradient Boosting.

**Figure 3:**
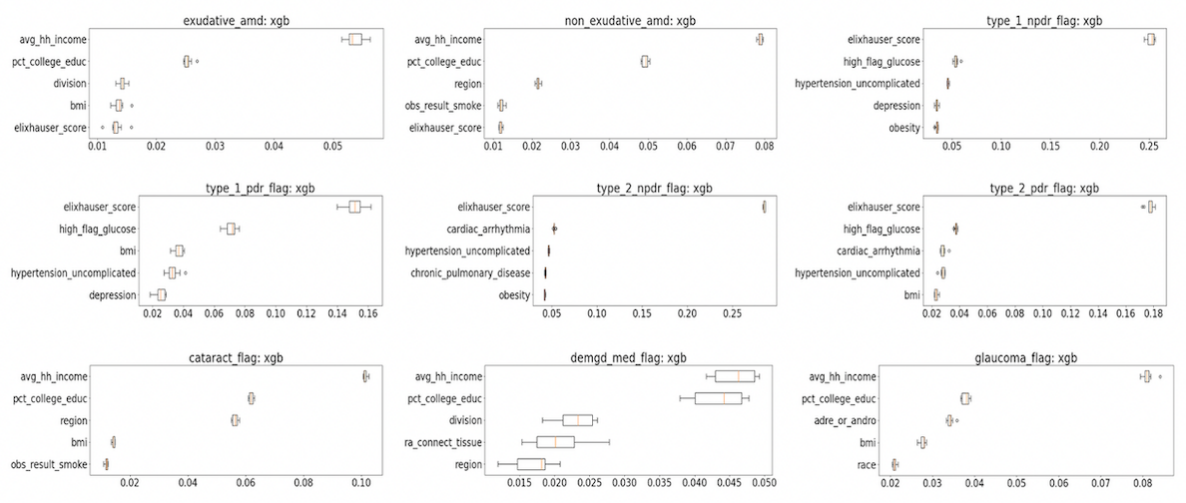
Clinical features primarily contributing to the predictions for each pathology. amd: age-related macular degeneration; demgd: dry eye and meibomian gland dysfunction; hh: household; npdr: nonproliferative diabetic retinopathy; pct: percentage; pdr: proliferative diabetic retinopathy; xgb: Extreme Gradient Boosting.

The performance of different ML strategies varied as well (Figures 1 and 2 and Table 4), but in all cases, XGBoost demonstrated the best performance, showing, respectively, a prediction accuracy and an AUC of 78.6% (95% CI 78.3%-78.9%) and 0.878 for visually significant cataract, 77.4% (95% CI 76.7%-78.1%) and 0.858 for exudative AMD, 79.2% (95% CI 78.8%-79.6%) and 0.879 for nonexudative AMD, 72.2% (95% CI 69.9%-74.5%) and 0.803 for OSD requiring medication, 70.8% (95% CI 70.5%-71.1%) and 0.785 for glaucoma, 85.0% (95% CI 84.2%-85.8%) and 0.924 for type 1 NPDR, 82.2% (95% CI 80.4%-84.0%) and 0.911 for type 1 PDR, 81.3% (95% CI 81.0%-81.6%) and 0.891 for type 2 NPDR, and 82.1% (95% CI 81.3%-82.9%) and 0.900 for type 2 PDR (Table 4). XGBoost identified several clinical attributes that were important for diagnosis prediction (Figure 3).

The top-performing models identified the following clinical and demographic features that were primarily contributing to the predictions for each pathology (Figure 3; continuous measures showed positive associations):

- Exudative AMD diagnosis prediction was associated, in order of importance, with average household income, percentage college education, geographical division (Middle Atlantic, East North Central, East South Central, New England, South Atlantic/West South Central, Mountain, West North Central, Pacific, other/unknown), the BMI, and the Elixhauser score (comorbidity index).
- Nonexudative AMD demonstrated similar associations. In order of importance, these were average household income, percentage college education, region (Northeast, Midwest, South, West, other/unknown), smoking, and the Elixhauser score.
- Glaucoma clinical associations, in order of importance, included average household income, percentage college education, adrenal or androgen use, the BMI, and race.
- Cataract clinical associations, in order of importance, included average household income, percentage college education, region, the BMI, and smoking.
- OSD associations, in order of importance, included average household income, percentage college education, geographical division, rheumatoid arthritis and connective tissue disease, and region.
- DR associations varied over different subpathologies but generally included the Elixhauser score, high serum glucose, the BMI, hypertension, chronic pulmonary disease, depression, cardiac arrhythmia, and obesity.

Performance in predicting the presence of pathology ranged from 71% in the case of glaucoma to 87% in the case of type 1 PDR, with an average performance of 80% across all groups. Since the intent was to identify at-risk patients, these performance values were used to determine disease odds ratios (ORs) according to the method described by Hogue et al [36].

Applying this to each of the models provided a clinically useful measure. The models identified patients with elevated ORs of the prevalence of pathology from 2.4 in the case of glaucoma to 5.7 in the case of type I NPDR, with an average OR of 3.9 (Table 5).

**Table 3:** Demographic information of each cohort with ocular disease. For each cohort, a control (age- and gender-matched) population of similar size was generated, without the condition of interest.

Characteristic		Exudative AMD^a^ (n=32,072)	Nonexudative AMD (n=114,839)	Cataract (n=197,570)	OSD^b^ requiring medication (n=3720)	Glaucoma (n=192,727)	Type I NPDR^c^ (n=20,654)	Type I PDR^d^ (n=4465)	Type II NPDR (n=155,927)	Type II PDR (n=21,032)
Age (years), mean (SD)		79.8 (10.4)	77.1 (10.7)	69.7 (9.9)	68.3 (14.0)	72.4 (13.3)	51.5 (16.0)	52.1 (14.6)	64.4 (12.9)	61.6 (12.7)
Gender (female), n (%)		19,885 (62.0)	70,971 (61.8)	115,183 (58.3)	3050 (82.0)	108,698 (56.4)	10,203 (49.4)	2170 (48.6)	77,028 (49.4)	10,032 (47.7)
**Race, n (%)**										
	Asian	353 (1.1)	1608 (1.4)	3951 (2.0)	52 (1.4)	3662 (1.9)	186 (0.9)	31 (0.7)	4054 (2.6)	484 (2.3)
	Black	374 (2.1)	2756 (2.4)	13,632 (6.9)	272 (7.3)	30,065 (15.6)	2231 (10.8)	545 (12.2)	24,948 (16.0)	3912 (18.6)
	White	27,903 (87.0)	97,843 (85.2)	160,229 (81.1)	3281 (88.2)	139,342 (72.3)	16,337 (79.1)	3393 (76.0)	106,342 (68.2)	13,166 (62.6)
	Unknown	3143 (9.8)	12,632 (11.0)	23,511 (11.9)	112 (3.0)	19,658 (10.2)	1900 (9.2)	500 (11.2)	20,582 (13.2)	3449 (16.4)
**Ethnicity, n (%)**										
	Hispanic	513 (1.6)	2067 (1.8)	5927 (3.0)	86 (2.3)	7516 (3.9)	888 (4.3)	223 (5.0)	13,722 (8.8)	2608 (12.4)
	Non-Hispanic	27,774 (86.6)	96,465 (84.0)	168,132 (85.1)	3553 (95.5)	164,589 (85.4)	17,804 (86.2)	3764 (84.3)	124,118 (79.6)	15,900 (75.6)
	Unknown	3784 (11.8)	16,307 (14.2)	23,511 (11.9)	82 (2.2)	20,622 (10.7)	1962 (9.5)	478 (10.7)	18,088 (11.6)	2524 (12.0)
Education (college educated), n (%)		7761 (24.2)	27,906 (24.3)	47,614 (24.1)	868 (23.2)	47,411 (24.6)	4936 (23.9)	1058 (23.7)	37,111 (23.8)	4943 (23.5)
Size of control population, n		32,072	114,839	197,570	3720	192,727	20,654	4465	155,927	21,032
Total population for modeling (cohort+control), n		64,144	229,678	395,140	7440	385,454	41,308	8930	311,854	42,064

^a^AMD: age-related macular degeneration.

^b^OSD: ocular surface disease.

^c^NPDR: nonproliferative diabetic retinopathy.

^d^PDR: proliferative diabetic retinopathy.

**Table 4:** Model accuracy, AUC^a^, sensitivity, and specificity.

Outcome and algorithms		Accuracy (95% CI)	AUC (95% CI)	Sensitivity	Specificity	
**Cataract**						
	XGBoost^b^	78.6% (78.3%-78.9%)	0.878 (0.875-0.880)	0.796	0.776	
	RF^c^	72.1% (71.8%-72.4%)	0.811 (0.808-0.814)	0.749	0.693	
	LR-L1^d^	68.9% (68.6%-69.2%)	0.767 (0.764-0.771)	0.683	0.695	
	LR^e^	68.9% (68.6%-69.2%)	0.767 (0.764-0.771)	0.683	0.695	
	J48 DT^f^	66.5% (N/A^g^)	0.710 (N/A)	0.702	0.628	
**Exudative AMD^h^**						
	XGBoost	77.4% (76.7%-78.1%)	0.858 (0.851-0.863)	0.769	0.778	
	RF	73.0% (72.2%-73.8%)	0.817 (0.810-0.825)	0.745	0.715	
	LR-L1	71.8% (71.0%-72.6%)	0.794 (0.786-0.802)	0.716	0.720	
	LR	71.8% (71.0%-72.6%)	0.794 (0.786-0.801)	0.717	0.720	
	J48 DT	68.1% (N/A)	0.721 (N/A)	0.707	0.660	
**Nonexudative AMD**						
	XGBoost	79.2% (78.8%-79.6%)	0.879 (0.876-0.882)	0.801	0.783	
	RF	73.3% (72.9%-73.7%)	0.823 (0.820-0.827)	0.768	0.698	
	LR-L1	71.3% (70.9%-71.7%)	0.794 (0.790-0.798)	0.729	0.697	
	LR	71.3% (70.9%-71.7%)	0.794 (0.790-0.798)	0.727	0.700	
	J48 DT	68.1% (N/A)	0.725 (N/A)	0.741	0.622	
**OSD^i^**						
	XGBoost	72.2% (69.9%-74.5%)	0.803 (0.780-0.824)	0.708	0.735	
	RF	70.9% (68.6%-73.2%)	0.771 (0.747-0.795)	0.749	0.669	
	LR-L1	69.0% (66.7%-71.3%)	0.757 (0.732-0.782)	0.691	0.688	
	LR	69.5% (67.2%-71.8%)	0.757 (0.733-0.782)	0.688	0.702	
	J48 DT	65.1% (N/A)	0.702 (N/A)	0.675	0.628	
**Glaucoma**						
	XGBoost	70.8% (70.5%-71.1%)	0.785 (0.782-0.788)	0.689	0.728	
	RF	67.9% (67.6%-68.2%)	0.741 (0.738-0.745)	0.656	0.702	
	LR-L1	61.8% (61.5%-62.1%)	0.669 (0.665-0.673)	0.622	0.614	
	LR	61.8% (61.5%-62.1%)	0.669 (0.665-0.673)	0.619	0.617	
	J48 DT	62.0% (N/A)	0.647 (N/A)	0.647	0.593	
**Type I NPDR^j^**						
	XGBoost	85.0% (84.2%-85.8%)	0.924 (0.919-0.930)	0.850	0.850	
	RF	79.5% (78.6%-80.4%)	0.872 (0.864-0.879)	0.799	0.790	
	LR-L1	83.5% (82.7%-84.3%)	0.908 (0.902-0.915)	0.847	0.824	
	LR	83.5% (82.7%-84.3%)	0.908 (0.902-0.915)	0.847	0.824	
	J48 DT	73.8% (N/A)	0.796 (N/A)	0.756	0.721	
**Type I PDR^k^**						
	XGBoost	82.2% (80.4%-84.0%)	0.911 (0.897-0.924)	0.816	0.828	
	RF	77.3% (75.4%-79.2%)	0.861 (0.846-0.878)	0.802	0.744	
	LR-L1	81.2% (79.4%-83.0%)	0.895 (0.881-0.910)	0.847	0.777	
	LR	80.8% (79.0%-82.6%)	0.894 (0.880-0.910)	0.829	0.787	
	J48 DT	72.4% (N/A)	0.804 (N/A)	0.761	0.686	
**Type II NPDR**						
	XGBoost	81.3% (81.0%-81.6%)	0.891 (0.888-0.893)	0.845	0.782	
	RF	75.1% (74.8%-75.4%)	0.833 (0.830-0.836)	0.751	0.752	
	LR-L1	79.1% (78.8%-79.4%)	0.866 (0.863-0.869)	0.843	0.739	
	LR	79.1% (78.8%-79.4%)	0.866 (0.863-0.869)	0.844	0.739	
	J48 DT	69.6% (N/A)	0.742 (N/A)	0.635	0.757	
**Type II PDR**						
	XGBoost	82.1% (81.3%-82.9%)	0.900 (0.893-0.907)	0.841	0.801	
	RF	77.7% (76.8%-78.6%)	0.858 (0.850-0.865)	0.763	0.790	
	LR-L1	79.9% (79.0%-80.8%)	0.880 (0.873-0.887)	0.834	0.763	
	LR	80.0% (79.1%-80.9%)	0.880 (0.873-0.887)	0.847	0.753	
	J48 DT	71.1% (N/A)	0.774 (N/A)	0.674	0.748	

^a^AUC: area under the curve.

^b^XGBoost: Extreme Gradient Boosting.

^c^RF: random forest.

^d^L1-LR: L1-regularized logistic regression.

^e^LR: logistic regression without regularization.

^f^DT: decision tree.

^g^N/A: not applicable.

^h^AMD: age-related macular degeneration.

^i^OSD: ocular surface disease.

^j^NPDR: nonproliferative diabetic retinopathy.

^k^PDR: proliferative diabetic retinopathy.

**Table 5:** Model accuracy and ORs^a^ by pathology.

Pathology	Model accuracy, %	OR (95% CI)
Exudative AMD^b^	77	3.4 (3.2-3.7)
Nonexudative AMD	79	3.8 (3.6-4.0)
Cataract	79	3.7 (3.6-3.8)
OSD^c^	72	2.6 (2.1-3.3)
Glaucoma	71	2.4 (2.4-2.5)
Type I PDR^d^	82	4.6 (3.6-5.9)
Type I NPDR^e^	85	5.7 (5.0-6.4)
Type II PDR	82	4.6 (4.1-5.1)
Type II NPDR	81	4.3 (4.2-4.5)

^a^OR: odds ratio.

^b^AMD: age-related macular degeneration.

^c^OSD: ocular surface disease.

^d^PDR: proliferative diabetic retinopathy.

^e^NPDR: nonproliferative diabetic retinopathy.

## Discussion

### Principal Findings

A major challenge of current deep learning (DL) models is that their training requires a large amount of data because insufficient data may decrease the performance of DL models [37]. The original EHR data pool for this study comprised more than 80 million patients, one of the largest AI projects of its kind in ophthalmology. The final study populations totaled 1,486,078 patients, 50% of whom were controls. In addition to the substantial patient population, this study examined 9 subpathologies using 5 different analytical modeling approaches to identify the most predictive model for each pathology.

The goal of this effort was to create a digital health tool to identify patients at higher risk for the presence of ophthalmic pathology and to do this based solely on the sort of non-ophthalmic data to which a PCP would have access. The authors do not propose to either make definitive ophthalmic diagnoses or predict the development of future pathology. Rather, this work seeks to identify patients whose clinical and demographic context is associated with the presence of AMD, cataract, clinically significant DR, glaucoma, or OSD of a magnitude requiring pharmacological therapy. The creation, demonstration, and real-world validation (within a clinical setting) of a deployable digital tool will be the next step of this project.

The application of such a model in the clinical setting would allow a PCP to identify patients nearly 4 times more likely to have ophthalmic pathology. Such a tool would bring a substantial benefit in the triage and referral of at-risk patients to eye care professionals.

### Data and Outcome Engineering

These data consist of diagnostic and procedure codes; biometric data, such as the BMI and vital signs; demographic information, including socioeconomic and geographical information; laboratory results; and medications prescribed. This information does not include the physician notes that might provide a rationale for the diagnoses recorded. Indeed, since only a limited number of diagnoses may be listed on a claim, it is possible that some extant diagnoses may have gone unrecorded. However, diagnoses like cataract and OSD may be overrepresented since the ICD-10 taxonomy does not distinguish between clinically significant cataract and OSD from cases in which these pathologies are subclinical. Indeed, it would be of little clinical utility to build an AI model that detects subclinical cataracts.

Ours is not the first study to be faced with the challenge of identifying clinically relevant diagnoses from large data sets. A 2018 study [38] investigated the precision of ICD-10 codes for patients with uveitis and found that 13 of 27 uveitides were imprecisely defined and that multiple codes were used to describe the same pathology. A 2020 study of ocular pathology in patients with stroke [39] noted fewer patients with glaucoma than anticipated and attributed this to the lack of ophthalmology clinic data. The authors noted that patients may be on glaucoma medications without a concurrent ICD-10 code recorded for glaucoma, suggesting that a diagnosis of glaucoma may have been recorded in the patients’ medical records before incorporation into the data set. The authors sought, therefore, to define the glaucoma cohort as those patients who met 1 or more of 3 criteria: an ICD-10 code of H40.1% (open-angle glaucoma), the prescription of glaucoma medication, or the presence of a CPT code indicating glaucoma surgery (see Tables 1 and 2). This definition was developed to both detect glaucoma patients without glaucoma ICD-10 codes and to exclude patients inappropriately labeled as glaucoma by ICD-10. This definition resulted in a substantial winnowing of the glaucoma cohort from 1,368,700, 50% of whom were controls, to 385,514 patients.

The authors took a similar approach to the cataract and OSD study populations. Cataract and OSD are among the most frequently recorded diagnoses on claims [40]. Cataract, in particular, is nearly ubiquitous in elderly patients and was the most common ophthalmic ICD-10 diagnosis of those examined here. Since only a subset of these patients require cataract surgery, the detection of cataract alone is not clinically useful. ICD-10 coding does not distinguish between cataracts requiring surgery and those that do not. However, CPT coding, in a sense does make this distinction. Therefore, CPT codes of 66984 (cataract extraction with intraocular lens) and 66982 (complex cataract extraction) were chosen as the criteria for clinically significant cataracts. This narrowing of the inclusion criteria reduced our cataract study population from 2,087,836, 50% of whom were controls, to 395,140 patients. OSD coding is even more problematic. A large number of ICD-10 codes are available, and clinical significance is difficult to establish. Our initial cohort of OSD patients and controls totaled 1,182,912 patients. To model the clinical context associated with OSD, a restrictive criterion was chosen: the prescription of topical cyclosporine or lifitegrast. This greatly reduced the OSD population to 7440 patients, but this ensured the final population represented patients with clinically meaningful disease. No outcome engineering measures were applied to the AMD groups or to the DR groups, each of which was defined by its corresponding ICD-10 code.

In addition, PDR and NPDR could have been combined into 1 group since the referring physician probably would not care about what sort of DR the patient has. However, the NPDR group is so much larger than the PDR group that the authors do not expect that the segmentation is detrimental.

### Clinical and Demographic Attributes and Feature Engineering

The initial data set included a large number of attributes or “features” (in the language of ML), totaling 380 individual parameters. To produce models that would not be burdensome for the clinician to use, the authors sought to reduce the number of attributes required by each model. This reduction and modification of model parameters is referred to as “feature engineering.” For a feature to be included in the final model, several criteria needed to be met. The feature must play a significant role in the model’s outcome. It is self-evident that features that do not contribute substantially to a model may be discarded with little impact on model performance. In the case of the XGBoost models, parameter optimization was performed by the grid search algorithm [41]. The second feature inclusion criterion was noncorrelation with other features. In some cases, such as between weight and the BMI, the correlation is evident. However, the correlation between other clinical features only becomes clear on analysis. The issue of feature correlation highlights a difference between AI and traditional risk analysis studies. When studied individually, certain attributes, such as obesity and socioeconomic status, may be identified as disease risk factors. However, when viewed collectively, the importance of 1 of these may be reduced if the 2 attributes are highly correlated. The third criterion for feature inclusion was high frequency in the data set. Some of the laboratory values, particularly serum fibrinogen, were so sparse in the data set that exclusion of the feature was preferable to the alternatives of sample reduction or interpolation. Two thresholds for feature sparsity were established in this project. Models were built upon data sets that excluded features with more than 20% missing values. Feature engineering substantially benefits from guidance by clinical domain experts [42], and our feature and outcome engineering was clinically informed, particularly in the realm of the diagnostic criteria described earlier. The features included in the final XGBoost model, the top-performing strategy, are available as supplementary materials to this manuscript. XGBoost is a DT-based ensemble modeling method. It can effectively capture the nonlinear relationship between predictors and the outcome by combining many weaker models to create a strong model. “Weak” and “strong” here refer to how correlated the models are to the outcome. The algorithm added models sequentially, and the next model corrected the error from the previous model. Through this iterative process, the data can be eventually accurately predicted by the model.

### Usage Data and Generalizability

The application of usage data to this effort is both a weakness and a strength of this project. These data do not contain the richness of a complete medical record. It is therefore impossible to establish the criteria under which the clinicians made the diagnoses recorded—hence our outcome engineering maneuvers to establish stricter criteria (eg, using CPT codes for cataract surgery to identify patients with clinically significant cataract). At the same time, models built upon these sorts of data are more generalizable and available than models built upon more specific and perhaps more idiosyncratic data sources. These are precisely the sorts of data available to PCPs, making these models more easily deployable than models built upon a specific medical record system. Indeed, the availability of these data is illustrated by our being able to investigate a base of more than 80 million patients from disparate health care systems.

Definitions of the parameters used in these models is a topic worth addressing. The parameters ingested by the models that are used to make predictions include pathologies and demographics that would ordinarily require a clear and consistent definition. These parameters include macular degeneration whose definition should be established a priori to demographic terms, such as gender and sex, that not only require definition but also incorporate the idea of nonbinary values.

It is the nature of large electronic medical record studies that such definitions are impossible to impose externally and that the interpretations of gender, hypertension, diabetes, and glaucoma are likely to vary among the practitioners and patients who themselves may be the source of the data of these values in the data set. Our use of a database of 80 million patients provides a large degree of protection from selection bias. However, because these clinical definitions are intrinsic to the data set itself, a great deal of caution must be exercised when attempting to draw inferences about pathogenesis simply by evaluating the most correlative features of the model. However, the limitation of the model to revealing the disease process makes the model no less valuable in its ability to predict which patients are at the highest risk for unrecognized eye disease.

### Hierarchical Relationships

It should be noted that the clinical features identified as relevant by each of the pathology models should be viewed as correlative but not necessarily causative. It is better to think of the collection of clinical values as a patient’s *clinical milieu* rather than as a collection of individual risk factors. Although it is difficult to imagine that college education is itself a risk factor for pathology, its correlation and importance to a given model should not be discounted, since it does contribute to the model’s predictiveness of the presence of pathology. All of this is not to say that causation may not exist in the relation between some of these features and the pathologies modeled. Highly multidimensional clinical AI studies like this one may identify previously unrecognized factors that directly influence pathogenesis. However, causative connection cannot be established by this sort of study and would require a more traditional experimental approach. Although the J48 DT models did not perform as well as the GLM or XGBoost strategies, they are informative in that they describe hierarchical relationships among clinical features. As an example, the J48 model for glaucoma identifies race, systemic steroids, and antidiabetic medication use as important clinical features. However, the model dictates the order in which these factors should be considered, assessing race only after it is established whether the patient takes antidiabetic medications and assessing systemic steroid use only after these first 2 attributes have been determined. Such a hierarchical relationship among clinical features and demographic characteristics would be enormously difficult to establish in traditional reduced-dimensional scientific queries. This gestalt approach to multidimensional clinical context is one of the strengths of AI.

### Decision Support

Ophthalmology is well suited for AI, given the rich visual information and data available; complex ophthalmological systems are better understood and eye care enhanced through sophisticated analysis and prediction. Integrating AI into clinical practice may facilitate better patient outcomes, given the complexity of disease diagnosis, treatment selection, and clinical testing. Ophthalmological clinical decision support systems that aid in diagnosis could improve the accuracy and efficiency of decision-making processes in ophthalmology, ultimately leading to improved patient access, outcomes, and potentially costs [43].

These models predict the presence of extant pathology. They would be of value in the identification of populations in which these pathologies are substantially more prevalent than in the general population. The models should not be used to make a diagnosis for an individual patient but rather to identify patients at risk of having undetected AMD, cataract, DR, glaucoma, or OSD. Further, these models are built upon clinical data in which an ophthalmic pathology is or is not present. That is to say, the models presented here are not constructed to predict the development of future pathology. It may or may not be the case that a particular clinical context, as defined by the multidimensional features incorporated into the models, may predict the development of future disease, but that is not appropriate way to use the models presented. These models predict the presence of ophthalmic pathology based upon non-ophthalmic data and would be best used for triage and referrals from non-ophthalmologists to eye care specialists. The research is designed to raise awareness about the variables associated with referral to heighten PCPs’ vigilance to the clinical and demographic characteristics that may need further reflection and attention.

### Real-World Application Prospects of Ophthalmological AI Models

Advances in computing power combined with disruptions in health care resulting from unprecedented circumstances of the COVID-19 pandemic have prompted the worldwide exploration of AI-based systems in several medical subfields, including ophthalmology [44]. Ophthalmology has been at the forefront of AI research, in particular ML and DL approaches, because of the ubiquitous availability of noninvasive, rapid, and relatively inexpensive ophthalmic imaging [45]. Ophthalmic AI systems are advantageous in that they decrease the amount of time required to interpret image data, enable ophthalmologists to gain a greater understanding of disease progression, and assist with early-stage diagnosis, staging, and prognosis [46].

Numerous factors will determine the successful adoption of AI technologies into clinical practice. AI innovations that help clinicians manage the complexity (rather than add yet another layer of complexity) associated with effective ophthalmological care will likely be better received. In addition, the ability for critical appraisals by optometrists and ophthalmologists will be key to validating the theoretic models. AI models can be difficult to interpret and explain, which can make it difficult for stakeholders to understand how decisions are made [47]. It is important that the AI models be transparent and explainable in order to gain and maintain the trust of health care professionals, patients, and other decision makers. Providers of AI technologies and educators also need to ensure that training needs are adequately assessed and value to patient outcomes demonstrated if the promise of AI in ophthalmological care is to be realized.

AI has the potential to provide invaluable insights across multiple domains of ophthalmology. By leveraging ML algorithms, AI can process and analyze vast amounts of information, including physiological data, EHRs, 3D images, radiology images, histologic evaluation, genomic sequencing, and administrative and billing data. One advantage that could be realized by the algorithms discussed herein is that they use commonly collected data contained within an EHR system to identify eye disease risk. This means that the algorithms could be deployed in the background of an EHR to enable inference of an entire PCP’s or practice’s patient population. The results of this inference could appear as a flag in a patient chart, alerting the PCP for a given patient as to the need to refer to an eye care professional for further evaluation. The approach of deploying these algorithms within the EHR would also enable further validation and assessment of algorithm generalizability prior to clearing the algorithm for regular use by PCPs. Additional validation steps such as this would help identify any local biases for a given patient population and enable monitoring performance for algorithmic drift.

Data infrastructure is an important influencer for the adoption of AI innovations. AI requires a continuous supply of high-quality data. Data quality issues may entail accuracy, completeness, consistency, timeliness, integrity, relevance, data collection, preprocessing, management, data governance, and data labeling [47]. Storage challenges, processing challenges, data management challenges, data heterogeneity, data privacy and security, bias and representativeness, and data access are also data quality considerations [47]. An appropriate data infrastructure, including its maintenance and evolution over time, is a prerequisite for successful AI applications.

Management of eye health necessitates a multidisciplinary team with a dynamic flow of information between treating doctors [48]. Holley and Lee’s [4] qualitative research found that PCPs had poor communication with eye care providers and the PCPs desire changes in the current referral-to-eye-care system. Better communication between PCPs and eye care professionals, further implementation of EHRs, and increasing eye screening in primary care clinics were common themes. Moudgil et al [48] found that 80% of the physicians communicated with ophthalmologists sometimes, whereas only 10% ensured communication at all times. The information sought by the treating physicians from the ophthalmologists regarding their referral for ocular findings included severity, the grading of DR, other ocular changes, need for intervention, and the frequency of screening and follow-up based on changes observed.

Finally, ethical considerations call for AI systems to adhere to the principles of fairness and nondiscrimination [49,50]. Advances in modern medicine are sometimes stymied by the inability to translate evidence-based care to all patients [51]. Transparency of AI models is essential to be able to evaluate and ensure their relevance for diverse populations and the ability to translate the innovations to all settings of care.

### Limitations

Several limitations are inherent in the use of aggregated clinical data. Longitudinal data on patients are limited, and this, by extension, limits projects such as ours in their ability to predict the development of future pathology. Although the data set does derive information from EHRs, including Epic, Cerner, GE, and McKesson, the actual physicians’ notes are not available for analysis. Aggregated data also disproportionally represent hospital encounters and underrepresent outpatient visits [52]. Attempts to mitigate some of these deficiencies in the feature and outcome engineering methods are described before. A certain degree of circumspection should be exercised when applying this model more broadly to other databases that may have used different NLP protocols.

A challenge with deploying these models in their current form is that the richness of data (ie*,* number of parameters) to be input into the models must be balanced against the labor the clinician must expend entering them. The authors sought to reduce feature input without substantially affecting model predictive performance. The goal is to develop tools that will aid clinicians and reduce the number of undiagnosed serious ophthalmic conditions. Empirically based analyses such those presented here are exploratory and intended to generate insights worthy of subsequent investigation with different study designs and methods that are better suited for causal inference.

It is important to note that data quality and representativeness are a potential issue for ML model training from EHRs and other clinical databases. EHR data can be incomplete, inconsistent, or erroneous, given the nature of the data collection and documentation. EHR data can also be biased toward populations with better access to health care. Some of these issues (eg, access) are inherent to our health care system in general and are not specific to EHR data. Regardless of the source of the issue, it is important to note that models trained and tested on EHR data may not be generalizable to the larger population.

### Conclusion

In summary, this research demonstrates real patient triage potential by deploying AI strategies directly to PCP EHRs. In addition, based on the original data pool (more than 80 million patients), the final study population size (1,486,078 patients, 50% of whom were controls) and the 9 subpathologies using 5 different analytical modeling approaches, the authors believe this study to be one of the largest AI projects in ophthalmology.

## Appendix

Multimedia Appendix 1 Patient inclusion and exclusion criteria and attrition.

## Abbreviations

AI: artificial intelligence
AMD: age-related macular degeneration
AUC: area under the curve
DEMGD: dry eye and meibomian gland dysfunction
DL: deep learning
DR: diabetic retinopathy
DT: decision tree
EHR: electronic health record
GLM: generalized linear model
ICD-10: International Classification of Diseases, 10th Revision
LR: logistic regression without regularization
L1-LR: L1-regularized logistic regression
ML: machine learning
NLP: natural language processing
NPDR: nonproliferative diabetic retinopathy
OR: odds ratio
OSD: ocular surface disease
PCP: primary care practitioner
PDR: proliferative diabetic retinopathy
RF: random forest
XGBoost: Extreme Gradient Boosting
