# Supplementary material for: Machine Learning Methods Using Artificial Intelligence Deployed on Electronic Health Record Data for Identification and Referral of At-Risk Patients From Primary Care Physicians to Eye Care Specialists: Retrospective, Case-Controlled Study
Source: JMIR AI. 2024 Mar 12;3:e48295. doi: 10.2196/48295 (PMC11041486; doi:10.2196/48295)
Supplement: Multimedia Appendix 1 [file ai_v3i1e48295_app1.docx]

**Multimedia Appendix 1.** Patient inclusion and exclusion criteria and attrition.

| Criteria | Keeping only the patients with diagnosis codes like 'H3530%'/'H3531%'/'H3532%' OR 'H25%' OR 'E083%'/'E093%'/'E103%'/'E113%'/'E133%' OR 'H40%' OR 'H041%'/'H0288% AND records with 'ICD10' diagnosis code type  Removing records with (1) ‘Y’ as the problem list flag, which indicates a problem with the recorded data; (2) diagnosis status that states 'History of', which indicates patient’s history with the disease; and (3) NULL/empty encounter ID | | | | |
| --- | --- | --- | --- | --- | --- |
| Condition | AMD | Cataract | DR | Glaucoma | Dry Eye/MGD |
| Total Cases | 294,739 | 1,191,492 | 348,056 | 843,560 | 660,218 |
| Criteria | Removing patients with ‘Unknown’ as birth year | | | | |
| Condition | AMD | Cataract | DR | Glaucoma | Dry Eye/MGD |
| Total Cases | 294,718 | 1,191,418 | 348,048 | 843,491 | 660,184 |
| Criteria | Removing patients with date of birth after index diagnosis date | | | | |
| Condition | AMD | Cataract | DR | Glaucoma | Dry Eye/MGD |
| Total Cases | 294,716 | 1,191,416 | 348,044 | 843,486 | 660,182 |
| Criteria | Removing records with interaction date of the encounter greater than index diagnosis date | | | | |
| Condition | AMD | Cataract | DR | Glaucoma | Dry Eye/MGD |
| Total Cases | 294,081 | 1,188,218 | 347,503 | 841,546 | 659,353 |
| Criteria | Removing patients with age <15 on index diagnosis date | | | | |
| Condition | AMD | Cataract | DR | Glaucoma | Dry Eye/MGD |
| Total Cases | 293,981 | 1,187,779 | 347,262 | 833,341 | 652,748 |
| Criteria | Removing patients with age <15 on index diagnosis date | | | | |
| Condition | AMD | Cataract | DR | Glaucoma | Dry Eye/MGD |
| Total Cases | 293,981 | 1,187,779 | 347,262 | 833,341 | 652,748 |
| Criteria | Removing patients with diagnosis record of disease of interest prior to index diagnosis date | | | | |
| Condition | AMD | Cataract | DR | Glaucoma | Dry Eye/MGD |
| Total Cases | 260,724 | 1,119,285 | 296,756 | 747,646 | 622,815 |
| Criteria | Removing patients not continuously enrolled for 60 days in the baseline period | | | | |
| Condition | AMD | Cataract | DR | Glaucoma | Dry Eye/MGD |
| Total cases | 242,418 | 1,045,291 | 276,504 | 685,489 | 592,135 |
| Criteria | Removing patients with date of death before index diagnosis date | | | | |
| Condition | AMD | Cataract | DR | Glaucoma | Dry Eye/MGD |
| Total Cases | 242,112 | 1,044,495 | 276,199 | 684,876 | 591,797 |
| Criteria | Removing patients with ‘Unknown’ as gender | | | | |
| Condition | AMD | Cataract | DR | Glaucoma | Dry Eye/MGD |
| Total cases | 241,882 | 1,043,918 | 275,942 | 684,350 | 591,456 |
| Criteria | Segregating patients into subsets based on AMD subtype OR  Removing patients with cataract-related procedure or diagnosis in the baseline, or without cataract-related procedure and diagnosis in the follow-up OR  Segregating patients into different subsets based on DR subtype OR  Keeping only those patients who have had open angle glaucoma or have consumed a glaucoma-related medication or have had a glaucoma-related procedure in the follow-up OR  Keeping only those patients who consumed DEMGD-related medications in the follow-up | | | | |
| Condition | AMD | Cataract | DR | Glaucoma | Dry Eye/MGD |
| Total Cases | Exudative: 32,075  Non-exudative: 114,839 | 197,570 | Type I NPDR: 20,654  Type I PDR: 4,465 Type II NPDR: 155,927 Type II PDR: 21,032 | 192,757 | 3,720 |
